# Column-Free Purification Methods for Recombinant Proteins Using Self-Cleaving Aggregating Tags

**DOI:** 10.3390/polym10050468

**Published:** 2018-04-25

**Authors:** Yamin Fan, Jackelyn M. Miozzi, Samuel D. Stimple, Tzu-Chiang Han, David W. Wood

**Affiliations:** William G. Lowrie Department of Chemical and Biomolecular Engineering, The Ohio State University, Columbus, OH 43210, USA; fan.457@osu.edu (Y.F.); miozzi.5@osu.edu (J.M.M.); stimple.1@osu.edu (S.D.S.); tzuchianghan@gmail.com (T.-C.H.)

**Keywords:** non-chromatographic protein purification, aggregating tags, split intein, elastin-like polypeptide (ELP), β-roll tag (BRT17), self-cleaving tag

## Abstract

Conventional column chromatography processes to purify recombinant proteins are associated with high production costs and slow volumetric throughput at both laboratory and large scale. Non-chromatographic purifications based on selective aggregating tags have the potential to reduce costs with acceptable protein yields. A significant drawback, however, is that current proteolytic approaches for post-purification tag removal after are expensive and non-scalable. To address this problem, we have developed two non-chromatographic purification strategies that use either the elastin-like polypeptide (ELP) tag or the β-roll tag (BRT17) in combination with an engineered split intein for tag removal. The use of the split intein eliminates premature cleavage during expression and provides controlled cleavage under mild conditions after purification. These self-cleaving aggregating tags were used to efficiently purify β-lactamase (β-lac), super-folder green fluorescent protein (sfGFP), streptokinase (SK) and maltose binding protein (MBP), resulting in increased yields compared to previous ELP and BRT17-based methods. Observed yields of purified targets for both systems typically ranged from approximately 200 to 300 micrograms per milliliter of cell culture, while overall recoveries ranged from 10 to 85 percent and were highly dependent on the target protein.

## 1. Introduction

Thanks to progress in molecular biology, cell engineering and fermentation design, upstream expression titers of recombinant proteins have increased significantly over the past twenty years [1]. Despite this success, however, developing large scale and cost-effective purification methods for recombinant proteins remains a fundamental challenge [2]. At laboratory scale, affinity domains such as Protein A, poly-Histidine, Chitin Binding Domain (CBD) and Maltose Binding Protein (MBP), have been utilized extensively due to their high specificity and simple purification methods [3]. Although the corresponding affinity resins are commercially accessible and can achieve a high degree of purity, these methods are inherently limited by high resin cost and slow volumetric throughput when processing large volumes. Thus, an opportunity exists in the use of non-chromatographic methods, which have significant potential for expanding bioprocess throughput and reducing purification costs at laboratory scale and in commercial manufacturing.

Over the years, biopolymers have been employed to create inexpensive non-chromatographic purification methods with good selectivity and yields. In most cases, engineered biopolymers serve as purification tags, which can induce a tagged fusion protein to form highly selective aggregates under specific chemical or physical conditions. Reported tags include the elastin-like polypeptide (ELP), repeat-in-toxin (RTX) domain, and ELK16 [4,5,6,7]. In particular, the ELP tag induces aggregation of fusion proteins under elevated temperature or high salt concentrations [4,5], while the consensus RTX domain, referred to as the β-roll tag (BRT17), reversibly forms a β-roll structure and precipitates in the presence of calcium [7].

As with any other purification tag, an important limitation of these tags is the occasional need to remove the tag after the purification process. This step is especially important for large precipitation tags that are likely to impact the characteristics of the target protein, and is critical in order to avoid unwanted immune responses when the target is to be used as a therapeutic. Common laboratory practices for tag removal include the use of proteases such as Factor Xa, TEV Protease, and Rhimovirus 3C Protease [8,9,10]. However, proteases require an additional purification step to remove them after the cleavage reaction, and may cause non-specific cleavage of the target or leave a small “stub” of amino acids on the target proteins [11]. These aspects of proteolytic tag removal, along with the high cost of the enzymes at large scale, have effectively limited their use to laboratory scale.

Inteins have been engineered over the last two decades to provide flexible self-cleaving modules for tag removal. Inteins can be genetically encoded into the tagged target protein sequence to provide site-specific self-cleavage of the tag from the target protein on demand. Compared to proteases, inteins are therefore more cost-effective with respect to buffer cost and production cost, since they are produced with the target protein in the expression host, and do not have to be supplied exogenously [12]. Self-cleaving inteins that have been developed for tag removal can be put into two categories: full-length inteins and split inteins. The most commonly used full-length inteins include the ΔI-CM intein developed in our laboratories, and the *Sce* VMA intein developed by New England Biolabs as part the IMPACT-CN system. With these inteins, C-terminal intein cleavage can be induced by either a pH shift (in the case of ∆I-CM) or addition of a thiol reagent (in the case of the *Sce* VMA intein) [13,14]. To date, they have been applied in various expression systems, including *E. coli*, *Picha pastoris, Nicotiana tabacum* and *Spodoptera fruigiperda* [14,15,16]. A persistent problem in intein applications, however, has been uncontrolled premature cleavage during protein expression. This phenomenon greatly limits production yields and makes inteins impractical for processes that require prolonged expression, making them effectively useless for mammalian cell expression hosts. Although this problem has been persistent and difficult to solve with full-length inteins, which are active during expression, split inteins have the potential to provide a solution. These inteins are inactive when they are initially expressed, and can only exhibit cleavage activity after the two intein fragments are reassembled. Thus, split inteins have the potential to completely eliminate uncontrolled premature cleavage during expression, enabling the application of intein-based self-cleaving tags to a wider variety of expression systems.

To demonstrate the potential impact of combining split inteins with polymeric aggregation tags, we have developed non-chromatographic purification strategies using the ELP and BRT17 tags in combination with an engineered split intein derived from *Nostoc punciforme* for tag removal. Importantly, the intein has been engineered to exhibit highly pH-sensitive cleavage, which minimizes product loss during precipitation and wash steps while providing rapid cleavage of the tag from the purified target protein. As a proof of concept, four target proteins were purified successfully in this work, including β-lactamase (β-lac), super-folder green fluorescent protein (sfGFP), streptokinase (SK) and maltose binding protein (MBP).

## 2. Materials and Methods

### 2.1. Chemicals and Reagents

All chemicals were purchased from either Sigma Aldrich (St. Louis, MO, USA) or Thermo Fisher Scientific (Waltham, MA, USA), unless otherwise stated. All cloning enzymes were purchased from New England Biolabs (Ipswich, MA, USA). All oligonucleotides were synthesized by Sigma Aldrich (St. Louis, MO, USA).

### 2.2. Plasmid Construction

Primer sequences used for plasmid construction in this study are available in Table 1. Plasmids encoding target proteins, β-lactamase (β-lac), streptokinase (SK), super-folder green fluorescent protein (sfGFP) and maltose binding protein (MBP) tagged with C-fragment of the split intein (NpuC*) were constructed by overlap PCR, where unique restriction sites for NdeI and XhoI were designed at the 5′- and 3′- ends of the PCR products, respectively. Each PCR-amplified fusion protein gene was digested with NdeI and XhoI and ligated into pET21a(+). To create the ELP-tagged NpuN* construct, the NpuN* fragment was amplified using the EcoRI-NpuN*-F and XbaI-NpuN*-R primers (Table 1). The PCR product was digested with EcoRI and XbaI and then ligated into the previously reported pET-ELP backbone plasmid [4]. The pMAL-BRT17 plasmid was purchased from Addgene (Plasmid #45358). The NpuN* fragment was amplified using EcoRI-NpuN*-Forward and HindIII-His6-NpuN*-R primers. The PCR product was digested with EcoRI and HindIII then ligated into a previously constructed pMAL-BRT17-∆I-CM backbone to replace ∆I-CM.

### 2.3. Protein Expression

All protein expression experiments were performed in the *Escherichia coli* strain BLR (DE3). Transformed cells containing the expression plasmids were cultured in 5 mL Luria Broth (LB) media supplemented with 100 μg/mL ampicillin at 37 °C for 16 to 18 h. The cultures were diluted 1:100 (*v*/*v*) into Terrific Broth (TB) media supplemented with 100 μg/mL ampicillin in Thomson‘s (Oceanside, CA, USA) Ultra Yield™ Flasks. The cells were then grown at 37 °C for 3–4 h until OD_600_ reached to 0.6–0.8. Protein expression was then induced by addition of 0.5 mM (final concentration) isopropyl β-d-1-thiogalactopyranoside (IPTG) and allowed to continue at 16 °C for 24 h.

### 2.4. Lysis and Recovery

Cells were harvested by centrifugation at 6000× *g* for 10 min at 4 °C. The cell pellets were either stored at −20 °C or immediately resuspended in either low salt buffer (20 mM AMPD, 20 mM PIPES and 1 mM EDTA at pH 8.5) for ELP purification or wash buffer (20 mM AMPD, 20 mM PIPES and 200 mM NaCl at pH 8.5) for BRT17 purification. In each case, the cell pellet was resuspended in one tenth of its culture volume before lysis. The resuspended cultures were then sonicated for 10 cycles of 30 s sonication at a setting of 4–5 W, with 30 s on ice. The lysate was then clarified by centrifugation at 15,000 rpm for 15 min at 4 °C and the supernatant was recovered for purification of the targets.

### 2.5. ELP-Mediated Protein Purification

For each ELP purification procedure, 500 µL of ELP-NpuN* clarified lysate was mixed with a variable volume of NpuC*-POI clarified lysate according to the expression titer of the target protein. Ammonium sulfate was then added to the mixture to a final concentration of 0.4 M (using a 1:5 dilution of a 2 M ammonium sulfate stock solution) and the solution was incubated at 37 °C for 10 min. The sample was then centrifuged at 15,000 rpm for 6 min and the recovered pellet was re-dissolved in low salt buffer (20 mM AMPD, 20 mM PIPES and 1 mM EDTA at pH 8.5). Another round of precipitation was done to increase the purity by adding ammonium sulfate as before to a final concentration of 0.4 M, and the solution was again incubated at 37 °C for 10 min. The sample was then centrifuged again at 15,000 rpm for 6 min and the pellet was then dissolved in cleaving buffer (20 mM AMPD, 20 mM PIPES, 1 mM EDTA at pH 6.2). The sample was incubated in 37 °C for 5 h. After 5 h of cleavage, ammonium sulfate was added as before at a final concentration of 0.4 M to precipitate the ELP tag, and the sample was incubated at 37 °C for 10 min and centrifuged at 15,000 rpm for 6 min. The purified product was recovered in the supernatant.

### 2.6. BRT17-Mediated Protein Purification

For each purification, 750 µL of BRT17-NpuN* clarified lysate was thoroughly mixed with NpuC*-POI clarified lysate at a volume dependent on the expression titer of the target protein. Calcium chloride was added to the mixture with a final concentration of 25 mM (using a 1:80 dilution of a 2 M stock solution) and the sample was mixed by pipetting and allowed to sit at room temperature for 15 min. The sample was then centrifuged at 16,000× *g* for 5 min and the supernatant was discarded. The remaining pellet was resuspended in wash buffer (20 mM AMPD, 20 mM PIPES and 200 mM NaCl at pH 8.5). The sample was then centrifuged again at 16,000× *g* for 5 min and the pellet was then dissolved in cleaving buffer (20 mM AMPD, 20 mM PIPES, 200 mM NaCl, and 25 mM EGTA at pH 6.2). The sample was incubated at 37 °C for 5 h. After 5 h of cleavage, calcium chloride was added as before to a final concentration of 25 mM to precipitate the BRT17 tag. The sample was mixed by pipetting and allowed to sit at room temperature for 15 min, then centrifuged at 16,000× *g* for 5 min. The purified product was recovered in the supernatant.

### 2.7. Intein Cleavage Analysis

For cleavage analysis, 20 μL samples were taken at different time points during the incubation process. The cleavage reaction in each sample was stopped by adding 2× sodium dodecyl sulfate-polyacrylamide gel electrophoresis (SDS-PAGE) loading dye and heated at 98 °C for 10 min. The percent cleaved at each time point was estimated by scanning densitometry of the precursors and the product bands on SDS-PAGE gels using ImageJ (NIH). The percent cleaved was calculated to be the intensity of the cleaved product band divided by the sum of the intensities of the precursor and product bands.

### 2.8. Protein Quantification

Protein concentration was determined using a Bradford Assay with Bovine Serum Albumin (BSA) used as a standard. The standard curve was generated by using 1, 2, 4, 6, 8 and 10 μg BSA/mL water. Clarified lysate samples were diluted 1:10,000 and purified product samples were diluted 1:100 in water. The Bio-Rad (Hercules, CA, USA) Quick Start™ Bradford 1× Dye Reagent (Bio-Rad Catalog #5000205) was used and OD_595_ was measured after 5 min incubation using a Biotek (Winooski, VT, USA) Synergy 2 plate reader. All assays were performed in triplicate.

### 2.9. Green Fluorescent Protein

Diluted samples of 100 μL each were read in a Biotek Synergy 2 plate reader with an excitation wavelength of 485 nm and an emission wavelength of 528 nm. All assays were performed in triplicate.

### 2.10. β-Lactamase Activity Assay

β-lac activity was determined using a nitrocefin dye-based colorimetric assay, where nitrocefin changes color from yellow to red when hydrolyzed by β-lac. A 0.5 mM stock solution of nitrocefin was made by dissolving the appropriate amount of nitrocefin in dimethylsulphoxide (DMSO). Protein samples were diluted with PBS and added to a 96 well plate with the nitrocefin solution to give a final nitrocefin concentration of 100 μM. The mixture was incubated for 2 min at room temperature. After the color development, OD_492_ was read using a Biotek Synergy 2 plate reader. An OD_492_ of 0.53 corresponds to 100 nmol of hydrolyzed nitrocefin [17]. One unit of β-lac is defined as the amount of β-lac needed to hydrolyze 1 μmol of nitrocefin in one minute. All assays were performed in triplicate.

### 2.11. Streptokinase Activity Assay

The activity assay of SK is an endpoint chromogenic method based on the potency of converting plasminogen to plasmin. In this method, plasmin accelerates the hydrolysis of Chromogenix (Bedford, MA, USA) S-2251™ (Catalog #820332) and results in the formation of a yellow end-product that can be detected at an optical absorbance of 405 nm. Thus, plasminogen activation by SK can be quantitatively assayed using the synthetic chromogenic substrate. To perform the assay, the samples were diluted in 1× sample buffer (10 mM Tris-HCl pH 7.4, 0.1 mM NaCl, 1 mg/mL BSA) to a desired concentration. Substrate solution was prepared by adding 1 mL 0.5 M Tris-HCl pH 7.4 to 1 mL 3 mM S-2251 and 5 μL 10% Tween 20 and was prewarmed to 37 °C. Immediately before use, 45 μL of glu-plasminogen (1 mg/mL) was added to the substrate solution. To assay in a 96-well plate format, 60 μL of diluted SK sample was mixed thoroughly with 40 μL of substrate solution. A standard curve was generated by using recombinant SK over the range 10.0 IU/mL, 5 IU/mL, 2.5 IU/mL, 1.25 IU/mL, 0.675 IU/mL. The plate was then incubated at 37 °C for 20 min and the absorbance was measured at 405 nm [18]. All assays were performed in triplicate.

## 3. Results

### 3.1. pH Controllability of Aggragating Tags

Most commonly, the cleavage activity of inteins can be induced or accelerated by either thiol addition, a small shift in pH or increases in temperature [19]. While thiol-induced inteins are more tightly controllable, pH-induced inteins are more economical at large scale [12]. Thiol compounds may also disrupt the disulfide bonds in the target protein, which makes thiol-induced inteins unsuitable for a significant number of targets. In this study, we used a split intein previously engineered from the Nostoc punciforme DnaE split intein, which exhibits enhanced pH sensitivity in on-column purifications [20]. To confirm that the pH sensitivity is retained with precipitation tags, we carried out cleavage kinetics studies to evaluate the pH controllability of this intein in fusion to the aggregating tags ELP and BRT17.

**Figure 1:**
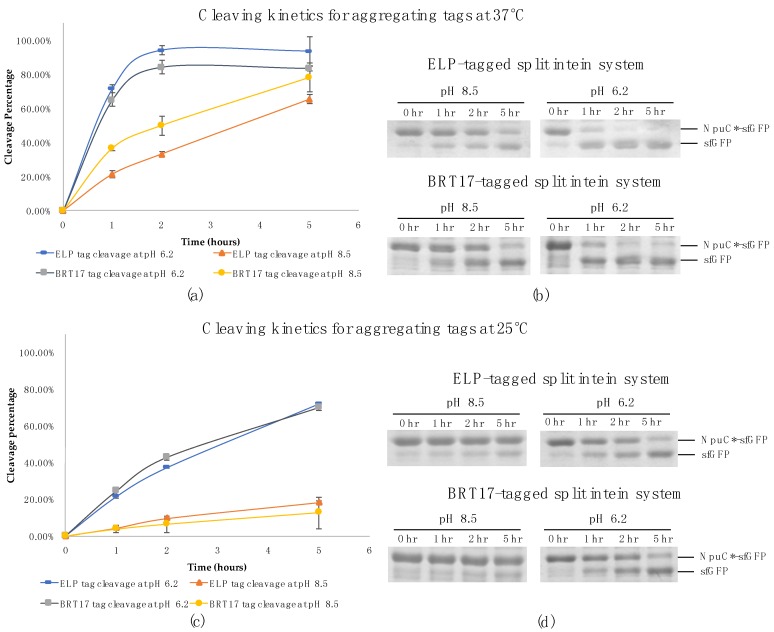
The cleavage kinetics of the ELP and BRT17-tagged split intein system for super-folder green fluorescent protein at pH 8.5 and pH 6.2 with different temperature conditions respectively (**a**) Normalized cleavage percentage of the reactions over time at pH 8.5 and pH 6.2 at 37 °C determined by ImageJ; (**b**) Samples at different time points (0 hr, 1 hr, 2 hr and 5 hr) during cleavage reaction at pH 8.5 and pH 6.2 at 37 °C on SDS-PAGE gels; (**c**) Normalized cleavage percentage of the reactions over time at pH 8.5 and pH 6.2 at 25 °C determined by ImageJ; (**d**) Samples at different time points (0 hr, 1 hr, 2 hr and 5 hr) during cleavage reaction at pH 8.5 and pH 6.2 at 25 °C on SDS-PAGE gels.

Time point samples were collected during the cleavage reactions for both self-cleaving aggregating tags at pH 8.5 and 6.2 respectively. At each time point the reactions were stopped by addition of 2× SDS-PAGE loading dye and heating for 10 min at 98 °C. With both tags and super-folder green fluorescent protein (sfGFP) as a model target protein, the cleavage reactions were almost complete after two hours of incubation at 37 °C in pH 6.2 buffer. Further, the final cleavage efficiencies were approximately 94% for the ELP system and 84% for the BRT17 system (Figure 1a,b). Importantly, the cleavage efficiency at pH 8.5 was approximately 2 to 4-fold lower at 37 °C and 4 to 6-fold lower at 25 °C, indicating that the pH sensitivity is retained with both tags (Figure 1). Specifically, at pH 8.5 and 37 °C, the cleavage efficiency was only 20% for ELP system and 35% for BRT17 system within the first hour of the reactions. Additionally, at pH 8.5 and 25 °C, the cleavage efficiency was only 4% for both systems after the first hour of the reactions. This controllability can be used to achieve minimum product loss during the initial precipitation and wash steps by performing them at room temperature, while providing rapid cleavage at 37 °C. In both cases, the initial precipitation and wash steps can be completed in less than an hour, potentially allowing the entire purification process to take place in less than 4 h.

### 3.2. Purification of Recombinant Proteins Using Self-Cleaving Elastin-Like Polypeptide (ELP) Tag

The ELP tag used in this work is comprised of 110 repeats of the block VPGXG, where X is Val, Ala, or Gly in a 5:2:3 ratio. In a previously developed ELP-based purification method from our lab, we used the ΔI-CM mini-intein derived from the Mycobacterium tuberculosis RecA intein as the self-cleaving element, and artificially split it into a 110-aa N-fragment and a 58-aa C-fragment [21]. In that work, both intein fragments were fused to ELP tags, while the C-fragment was also fused to the N-terminus of the target protein. The two fragments were expressed separately and could be either purified separately and mixed, or combined before cell lysis and co-purified in a single mixture, in both cases eliminating premature cleavage during expression. Although that method was successfully demonstrated, the affinity of the two intein fragments for each other and the cleavage activity of the artificial split intein were much lower than the contiguous ∆I-CM parent intein. More importantly, fusions to the intein fragments tended to form aggregates during expression, which is often observed with artificially split intein systems [22,23,24]. It has also been hypothesized that the ELP tag places a significant metabolic burden on the expressing cells, and therefore may decrease overall expression of the target protein in ELP fusions. The Npu intein of the current strategy is naturally split, however, and tends to exhibit much better folding in fusion to various target proteins, and has also exhibited rapid splicing and cleaving in some previously reported systems [25,26]. Because the Npu intein fragments exhibit better folding, the fusion between the intein C-fragment and the precursor protein no longer requires a solubility tag, which was the main function of the ELP tag in the ΔI-CM artificially split intein system. Instead, the 36-aa Npu C-fragment alone acts as a small affinity tag for the target protein, where it is captured by the aggregating ELP-intein N-fragment through quick and tight association. Consequently, the current design is likely to increase the final yields and extend the capability of the system to purify a wider range of target proteins.

**Figure 2:**
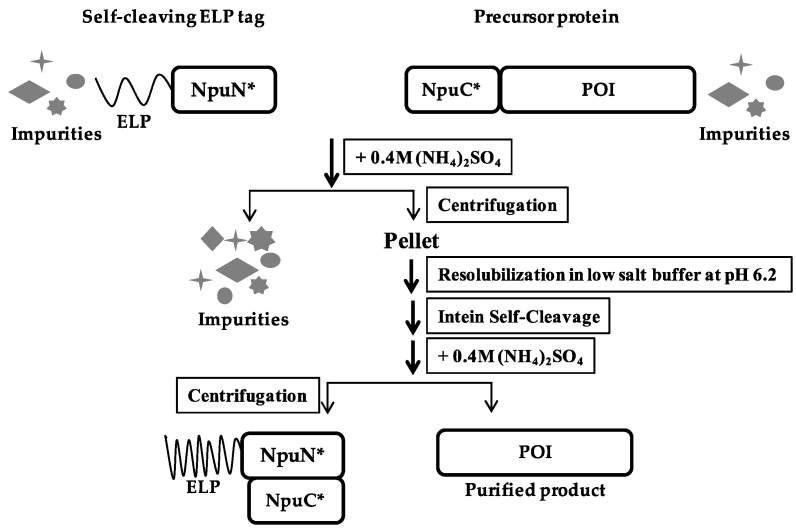
Schematic of ELP-tagged split intein purification method.

**Figure 3:**
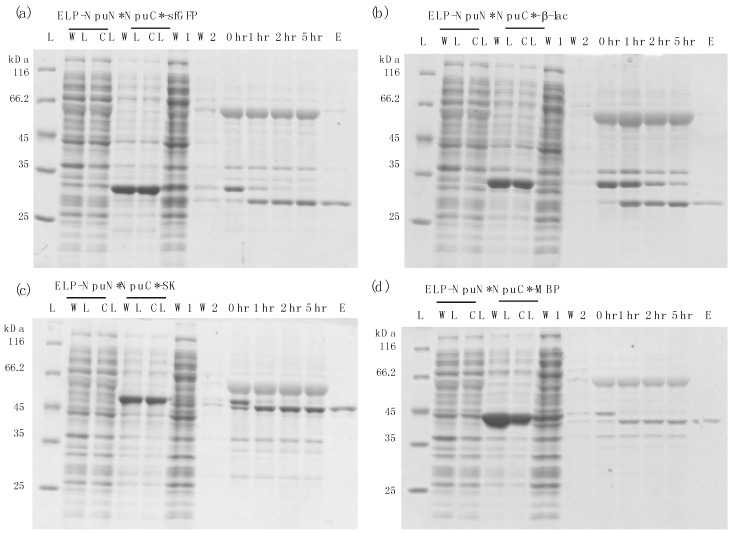
Purification results using ELP self-cleaving tag for different target proteins by Coomassie staining (**a**) super-folder green fluorescent protein (sfGFP); (**b**) β-lactamase (β-lac); (**c**) Streptokinase (SK); (**d**) Maltose-binding protein (MBP). Lanes: L: protein ladder; WL: whole lysate; CL: clarified lysate; W1: supernatant of first precipitation; W2: supernatant of second precipitation; 0 hr: start of cleavage reaction at pH 6.2, 37 °C; 1 hr: after 1 h of cleavage; 2 hr: after 2 h of cleavage; 5 hr: after 5 h of cleavage; E: elution sample (recovered protein in the supernatant).

A schematic of the purification method based on the self-cleaving ELP tag is shown in Figure 2. The self-cleaving aggregating tag (ELP-NpuN*) and the tagged target protein of interest (NpuC*-POI) were initially expressed separately and their clarified lysates were then mixed together to capture the target protein onto the ELP tag segment. Due to the strong affinity between the N and C-fragment of the *Npu* DnaE intein [27], both the aggregating tag and the precursor protein will be precipitated upon addition of 0.4 M ammonium sulfate and thus separated from the host cell proteins and impurities. The assembly of the two intein fragments and purification of the precursor proteins were performed in buffers at pH 8.5, after which the complex was solubilized in a low salt buffer at pH 6.2 to accelerate the cleavage rate. After 5 h of incubation at low pH, the cleaved ELP tag was separated from the product protein through another round of precipitation, allowing the eluted product to be collected in the supernatant. To demonstrate the general applicability of the ELP-tagged split intein method for purification, four target proteins of various sizes (super-folder green fluorescent protein, β-lactamase, streptokinase and maltose-binding protein) were purified (Figure 3). All four target proteins could be purified successfully using the self-cleaving ELP tag, where each showed no premature cleavage in vivo but fast cleavage upon dissolution in low pH buffer. The yield, specific activity and recovery of each target is summarized in Table 2 for each protein. All of them showed 4 to 8-fold increase in yields compared to the previous dual ELP-tagged split ΔI-CM purification system, ranging from approximately 200–300 μg per ml of shake flask culture.

### 3.3. Purification of Recombinant Proteins Using the Self-Cleaving β-Roll Tag (BRT17)

The BRT17 tag used for purification in this work consists of 17 repeats of a 9-aa monomer, GGXGXDXUX, where X is variable and U is a hydrophobic amino acid. This sequence is derived from the consensus block V RTX domain from the adenylate cyclase toxin (Cya A) of *B. pertussis* [7]. A key feature of this domain is that it reversibly self-assembles and becomes insoluble in the presence of relatively low concentrations of calcium ion. In a previously developed BRT17-based purification method from the Banta lab at Columbia University, four proteins were fused with BRT17 tag and purified by selective precipitation using differing concentrations of calcium chloride. During this work it was shown that the optimal concentration of calcium chloride for purification is 25 mM, where each purified protein was subsequently cleaved from the tag by enterokinase as a proof of concept. Since the BRT17 construct has not been used with an intein, we used the engineered Npu intein to study the efficiency of this purification process with a self-cleaving tag removal process.

**Figure 4:**
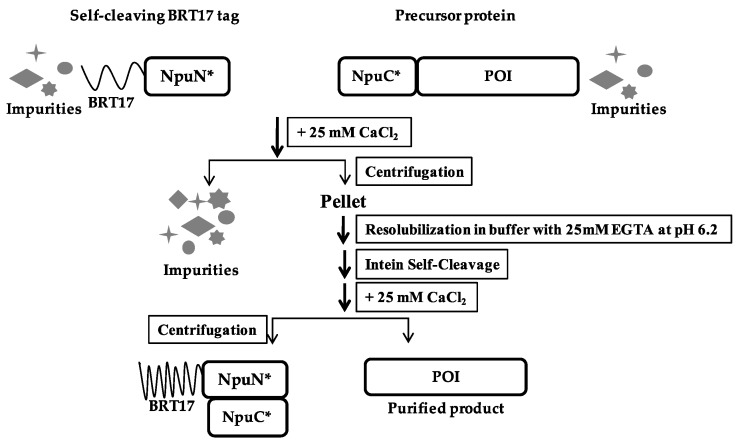
Schematic of BRT17-tagged split intein purification method.

**Figure 5:**
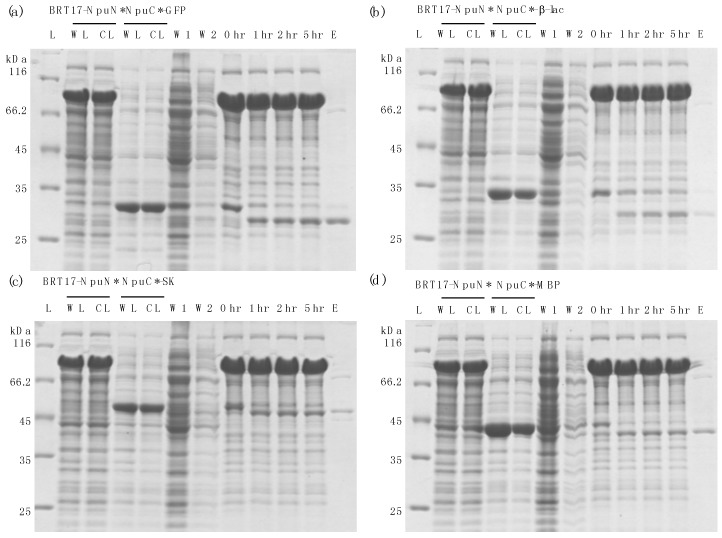
Purification results using ELP self-cleaving tag for different target proteins by Coomassie staining (**a**) super-folder green fluorescent protein (sfGFP); (**b**) β-lactamase (β-lac); (**c**) Streptokinase (SK); (**d**) Maltose-binding protein (MBP). Lanes: L: protein ladder; WL: whole lysate; CL: clarified lysate; W1: supernatant of first precipitation; W2: supernatant of second precipitation; 0 hr: start of cleavage reaction at pH 6.2, 37 °C; 1 hr: after 1 h of cleavage; 2 hr: after 2 h of cleavage; 5 hr: after 5 h of cleavage; E: elution sample (recovered protein in the supernatant).

Similar to ELP-based method described earlier, the N-fragment of the engineered *Npu* intein was fused with the BRT17 tag, while the C-fragment of the intein was added to the N-terminus of the protein to be purified (Figure 4). The self-cleaving aggregating tag and the precursor protein were expressed separately, and the clarified lysates were then mixed together to capture the target protein of interest onto the BRT17 tag. Both the BRT17 tag and the precursor protein were then precipitated upon addition of 25 mM calcium chloride, and thus separated from the host cell proteins and impurities. The precipitation and wash steps were performed in pH 8.5 buffer with 25 mM calcium chloride, after which the precipitated pellet was dissolved in pH 6.2 buffer with 25 mM EGTA to sequester the calcium ion. To demonstrate the general applicability of the BRT17-tagged split intein method for purification, the same four target proteins were chosen and purified, including sfGFP, β-lac, SK and MBP (Figure 5). Similarly, all four target proteins could be purified successfully by using the self-cleaving BRT17 tag, and the yield, specific activity and recovery of each protein were subsequently determined (Table 2). Although the yields of each target might be overestimated by Bradford assay due to the impurities in the elution samples (lanes E in Figure 4), both sfGFP and β-lac yields were reported to be 291.5 ± 78.0 μg/mL and 196.5 ± 27.1 μg/mL, which is equivalent to 10.3 ± 2.7 μM and 6.8 ± 0.9 μM. In the previously published paper by Shur et al., the final yields were equivalent to 4.0 ± 0.01 μM and 1.4 ± 0.03 μM respectively [7]. While SK and MBP have no previous published values using this aggregating tag, we can conclude that the current method resulted in higher yields as proven by sfGFP and β-lac.

## 4. Discussion

In this study, we report two non-chromatographic purification methods that utilize aggregating tags with an engineered split intein. In both methods, the self-cleaving aggregating tag brings the target protein into an insoluble phase, allowing it to be purified by simple centrifugation and washing. Subsequent cleavage of intein delivers a purified native target in the soluble phase for simple recovery. In addition, since each fragment of the split intein alone is incapable of self-cleaving, both methods eliminate premature cleavage completely in vivo, but still provide rapid and pH-sensitive cleavage in vitro during the purification process. Further, due to the small size of the C-fragment of the intein (~3.6 kDa), our methods generally provide higher yields compared to systems where tags were expressed in fusion to target proteins [7,21].

While the ELP purification strategy employs multiple cycles of phase transition mediated by salt concentration, the BRT17-mediated purification is achieved by calcium addition. Comparing the purification results by these two methods, higher purity and yield of the protein products were generally obtained by using the self-cleaving ELP tag. Particularly, the relatively low yield and specific activity of β-lac from the BRT17-mediated purification is very likely due to the reported instability of the protein in buffers containing calcium chloride [7]. This led to inhibited cleavage during incubation and partial insolubility of the protein during elution. Therefore, for purification of β-lac or other proteins that precipitate with addition of calcium solution, the ELP-mediated purification strategy could be employed instead to provide higher yield, purity and specific activity. Alternatively, for protein targets that are sensitive to high salt concentration, the BRT17-mediated purification strategy may be more desirable. Other reported systems using aggregating tags include ELK16, L_6_KD, FK and FR, which have also been fused to the contiguous ΔI-CM intein [28]. When expressed with target proteins, these peptides led to the formation of active aggregates and the intein cleavage could be used to recover the proteins in solution. Although these systems are very effective for purification of unstable peptides, they resulted in low yields due to premature cleavage by the ΔI-CM intein. Similar strategies described in this work could be adopted using the engineered pH-sensitive split intein for improving the purification results.

When comparing the cleavage rates of the chosen purified protein, it is clear that SK reported the fastest cleaving for both methods. It is also determined that β-lac showed the slowest cleaving rate. The observed differences in cleavage rate of different target proteins can be attributed to different C-exteins leading the target protein sequences, which have observed in several different systems [20,26]. Therefore, for specific targets, each method could be optimized by differing incubation time and/or temperature, possibly resulting in a shorter processing time or an improved yield. In some cases, making small changes to the initial amino acids of the target protein may also help to increase cleaving rate and controllability.

Both methods could be potentially applied to mammalian and other eukaryotic expression systems that require higher expression temperatures or extended expression times. The elimination of premature cleavage and the use of mild cleavage conditions enable the production and purification of proteins with disulfide bonds and important therapeutics with complex glycosylation patterns. In conclusion, the two methods reported in this work provide simple and cost-effective column-free purification process as alternatives to the current downstream chromatography purification.

## Figures and Tables

**Table 1:** Primer list.

Primer Name	Sequence 5′–3′
BglII-Forward	ATCGAGATCTGACGTCCGATCCC
NpuC*-Blac-1 (overlap)	CTCAAAAATGGCTTCATAGCTCATAATATGCACCCAGAAACGCTGGTG
NpuC*-Blac-2 (overlap)	CACCAGCGTTTCTGGGTGCATATTATGAGCTATGAAGCCATTTTTGAG
Blac-2 (His-Stop-XhoI)	GCGCTCGAGTCAGTGATGATGATGATGATGCCAATGCTTAATCAGTGAGGC
NpuC*-sfGFP-1 (overlap)	CAAAAATGGCTTCATAGCTCATAATATGGTGAGCAAGGGCGAGGAGCTG
NpuC*-sfGFP-2 (overlap)	CAGCTCCTCGCCCTTGCTCACCATATTATGAGCTATGAAGCCATTTTTG
T7 terminator	GCCCCAAGGGGTTATGCTAG
NpuC*-MBP-1 (overlap)	CTCAAAAATGGCTTCATAGCTCATAATATGAAAATCGAAGAAGGTAAA
NpuC*-MBP-2 (overlap)	TTTACCTTCTTCGATTTTCATATTATGAGCTATGAAGCCATTTTTGAG
MBP-2 (His-Stop-XhoI)	GCGCTCGAGTCATTAATGATGATGATGATGATGCGAGCTCGAATTAGTCTGCGC
EcoRI-NpuN*-F	GCGGAATTCGGTGACGGTCACGGTGCCTTAAGCTATGAAACGGA
XbaI-NpuN*-R	GGCTCTAGATTAATTCGGCAAATTATCAACCCG
BRT17-I-F	GACCTAGAGGAGTAATAATAATAACAATAACAACAACCTCGGGATCGAGGGAAGGATTTC
BRT17-I-R	GACCTAAAGCTTTTAGTTGTGTACAACAACCCCTTCGGC
EcoRI-NpuN* Forward	GAATTCGGTGGAGGCGGGTCTGGTGACGGTCACGGTGCCTTAAG
HindIII-His6-NpuN*-R	ATATAAGCTTATGGTGATGGTGATGGTGATTCGGCAAATTATCAACCCGC

**Table 2:** Summary of purification results by using two different self-cleaving aggregating tags.

Aggregating Tag	Product Protein	Yield ^1^ (μg/mL)	Specific Activity (Unit/mg)	Recovery ^2^
ELP	sfGFP	338.5 ± 45.5	Fluorescent under 485 nm excitation	28.6 ± 0.1%
	β-lactamase	248.1 ± 43.2	167.2 ± 13.3	79.0 ± 4.4%
	Streptokinase	389.0 ± 65.3	22600.5 ± 1851.9	30.3 ± 0.5%
	MBP	223.0 ± 62.2	Binds Maltose resin	NR ^3^
BRT17	sfGFP	291.5 ± 78.0	Fluorescent under 485 nm excitation	49.0 ± 8.9%
	β-lactamase	196.5 ± 27.1	84.1 ± 10.1	85.8 ± 7.8%
	Streptokinase	210.7 ± 40.8	5874.7 ± 2234.9	11.8 ± 5.1%
	MBP	214.0 ± 44.0	Binds Maltose resin	NR ^3^

^1^ Yield is defined as μg recovered protein of interest per mL shake flask culture by using 1 mL of N-part clarified lysate; ^2^ Recovery is based on activity for elution and total cell lysate; ^3^ Not reportable.
